# One-year mortality after hip fracture surgery and prognostic factors: a prospective cohort study

**DOI:** 10.1038/s41598-019-55196-6

**Published:** 2019-12-10

**Authors:** Mattia Morri, Elisa Ambrosi, Paolo Chiari, Antonella Orlandi Magli, Domenica Gazineo, Fabio D’ Alessandro, Cristiana Forni

**Affiliations:** 1 Servizio di Assistenza Infermieristica, Tecnica e Riabilitativa, Rizzoli Orthopedic Institute, Via Pupilli 1, 40136 Bologna, Italy; 2 Department of Medical and Surgical Sciences, University of Bologna, Via Massarenti 9, 40138 Bologna, Italy; 3 Evidence Based Nursing Centre, S. Orsola-Malpighi Teaching Hospital, Via Albertoni 15, 40138 Bologna, Italy

**Keywords:** Geriatrics, Risk factors

## Abstract

Older adult patients with hip fractures are 3–4 times more likely to die within one-year after surgery than general population. The study aimed to identify independent predictive factors associated with one-year mortality after hip fracture surgery. A prospective prognostic cohort study was performed. All patients aged ≥65 years, consecutively admitted in three Italian hospitals with a diagnosis of fragility hip fracture were included. Patients with periprosthetic or pathological fractures were excluded. Multivariate analysis was used to determine variables that significantly increased the risk of one-year mortality and Receiver operating characteristic (ROC) curve analysis to assess their predictive capacity on the outcome.1083 patients fulfilled the inclusion criteria and the one-year follow-up was reached in 728 patients. The 16.6% of patients died within one-year after surgery. At the multivariate analysis, advancing age (OR = 1.094, 95% CI = 1.057–1.132), higher baseline Charlson Index (OR = 1.257, 95% CI = 1.114–1.418) and Activities of Daily Living scores (OR = 1.259, 95% CI = 1.143–1.388), presence of hospital-acquired pressure ulcers (PUs) (OR = 1.579, 95% CI = 1.002–2.489) and lack recovery of ambulation (OR = 1.736, 95% CI = 1.115–2.703), were found to be independent predictive factors of one-year mortality after surgery. The area under the ROC curve of the model was 0.780 (CI95% 0.737–0.824) for one-year mortality in elderly hip fractures patients. Early ambulation and careful long-term follow-up, with attention to frailty in elderly people, should be promoted.

## Introduction

After the age of 65 hip fractures become a particularly burdensome event for patients, their families and for the health service^1–4^. They have been associated with increased morbidity, loss of autonomy in Activities of Daily Living (ADLs), high rate of institutionalization and mortality^4,5^. The mortality in the first year after hip fractures surgery is high, ranging between 15% and 36%^4,6–9^. Dubljanin-Raspopovic *et al*.^10^ showed that the mortality rates a year after hip fracture was 3–4 times higher than expected in the general population.

Advanced age, male sex, clinical comorbidities, place of residence before fracture, cognitive impairment and time-to-surgery have been suggested as significant predictors of postoperative mortality in hip fracture patients^6,8,11–18^.

At the same time, recent systematic reviews have shown the need for further good quality studies to better define hip fracture patients’ mid-long-term outcomes and determinants^19,20^.

Furthermore, there are very few studies that, besides variables related to patient’ characteristics and/or to surgical intervention, describe and analyse the relationship between mortality and possible postoperative variables. The acute postoperative phase is generally seen as a delicate moment of patient recovery that can provide clinicians with precious information about patients’ possible survival and their future level of autonomy^19^. Mariconda *et al*.^8^ reported delirium, pressure ulcers, and anaemia as the main postoperative complications during hospitalization, and highlighted a significant correlation with one-year mortality. Moreover, delayed recovery of ambulation during hospital stay has been associated with poorer outcomes upon discharge^21^ and reduced survival at six months^22^. This aspect has not been described in the literature in relation to a longer follow-up.

It is important to establish the weight of each perioperative variable to survival to inform clinical care. Therefore, this study aimed to identify independent predictive factors associated with one-year mortality after hip fracture surgery.

## Materials and Methods

### Study design, setting and sample

A prognostic cohort study involving three Italian public hospitals was conducted from October 2013 to October 2015. All patients aged 65 years or older, consecutively admitted to the Emergency Departments (EDs) of the involved hospitals with a diagnosis of fragility (osteoporotic) hip fracture (pertrochanteric, femoral neck and subtrochanteric) were enrolled in the study. Fragility hip fracture was defined as a hip fracture occurring after a minimal trauma, such as a fall from standing height or lower.

Exclusion criteria were the patient’s refusal to take part in the study or the absence of a legal representative for patients unable to provide consent for medical reasons, and a diagnosis of periprosthetic or pathological fracture.

In the involved hospitals, early surgery (within 48 hours from the trauma) has been guaranteed since the early 2000’s. After arriving at the ED, the patient is triaged, prioritized by the admissions nurse and transferred to an orthopedic surgery department or to an orthogeriatric department, depending on beds’ availability. The duty orthopedic surgeon carries out a physical examination and assessment and then establishes the surgical technique. The inpatient rehabilitation treatment, aiming at allowing an early verticalization and walking, is started the day after surgery. It consists of two physical therapy sessions a day for six days a week. After the postoperative hospitalization phase a different pathway is defined for each patient tailored to their rehabilitation and nursing needs. This pathway might involve discharge from hospital to a rehabilitation care home, nursing home, or home care. The choice of most appropriate setting and intensity of treatment is made by an internal multi-professional team based on several elements, such as the patient’s clinical condition, cognitive status, social care network and orthopedic indication for weight bearing (early or delayed). During the period of the study, no changes in the care regime of hip-fracture patients were implemented.

For the cohort of included patients, we assessed different clinical outcomes: the primary outcome was the incidence of hospital-acquired pressure ulcers and their predictive factors, the secondary outcomes were 30-day and one-year mortality and their predictive factors. Results on short-term outcomes^23,24^ have been previously published; we here present the one-year follow up results.

### Variables and data collection

The outcome of the study was the mortality occurrence at one year after hip fracture surgery, which was assessed by a telephone follow-up.

Possible predictive factors for mortality were identified by a multi-professional team of experts, who selected the relevant variables based on clinical experience and data available in the literature^6,8,11–20^. To facilitate analysis and interpretation of data, three groups of variables were formed: those relevant to the patient’s preoperative status, those concerning surgical treatment, and those related to the postoperative period.

The preoperative variables, such as age (in years), gender, pre-fracture residential status (home or nursing home), type of hip fracture (pertrochanteric, femoral neck or subtrochanteric), number and location of any other fractures, pre-fracture activities of daily living independence (ADLs)^25^, presence of comorbidities (Charlson Index)^26^, physical condition (e.g. thin, normal, morbid obese according to Body Max Index (BMI), presence of pressure ulcers and haemoglobin (Hb) level, were collected within the first 24 hours of hospital admission by interviewing the patient or legal representative and clinical observation or by consulting medical records.

The variables related to surgery, such as time from arrival in Emergency Room (ER) to surgery, type of surgical procedure (e.g. arthro/endoprothesis or osteosynthesis) and length of surgery (in minutes) were obtained from the medical chart.

After surgery, the following variables were assessed on a daily basis at the bedside or through medical charts: the presence of pain (percentage of days when the NRS score or PAINAD score, for cognitive impaired patients, was above 4), of infections (urinary, pulmonary or from the surgical wound), of hospital-acquired pressure ulcers (PUs), of disorientation (clinical evaluation), of anaemia (rate of reduction in the haemoglobin level with respect to the level at presentation), lack recovery of ambulation and the presence of an informal caregiver at the patient’s bedside for at least half day. Moreover, the admission to an orthogeriatric ward, was assessed. The number of days of hospitalization was collected at patient’s discharge. A telephone follow-up was also performed one-year from surgery to assess patient’s survival and adherence to postoperative treatment after hospital discharge.

Data collectors were trained Registered Nurses (RNs) and physiotherapists with experience in hip fracture care and in clinical research, not involved in patient’s care, and independent from the researcher analysing the data.

### Sample size

The method for determining the sample size was based on Soper, D.S. (2017). A-priori Sample Size Calculator for Multiple Regression [Software]. available from http://www.danielsoper.com/statcalc). Simulation studies examining predictor variables for inclusion in logistic regression models suggest 10 events are necessary for each predictor, to avoid overfitting^27^. Considering the number of predictive parameters inserted into the multivariate analysis for the primary outcome [see^23^], it was estimated that at least 800 patients had to be included.

### Data analysis

The data collected were calculated using SPSS v.19.0 (IBM Corp., Armonk, NY, USA). All continuous data were expressed in terms of the mean and the standard deviation of the mean or median and interquartile range when not normally distributed; the categorical data were expressed as frequency and percentages. For the categorical variables collected daily during hospitalisation, the percentage of days with the presence/absence of the predictor in relation to number of days of hospitalisation was also calculated according to the following formula: percentage of days = (days/length of hospital stay) × 100. To assess the normal distribution and the homoscedasticity of the included variables, the Kolmogorov-Smirnov test and the Levene test were performed, respectively. Analysis of variance (ANOVA), Mann-Whitney test, Kruskal-Wallis test, Fisher exact test and Pearson Chi-square test were used for data analysis as appropriate. To determine which factors were independently associated with one-year mortality after hip fracture surgery, a logistic regression model using the Wald Backward method was performed. Variables with p-values ≤ 0.10 according to the univariate analysis and age (independent of p -value) were used as independent variables and entered into multiple logistic regression analyses, with the occurrence of one-year mortality as dependent variable.

In order to check the stability of the model, the logistic regression with bootstrap method with 100000 samples was performed three times. Moreover, to check the predictive value of the model, a receiver operator characteristic (ROC) curve analysis was carried out. P < 0.05 was considered significant for all tests.

### Ethics

The study was approved by the ethics committee of the three participating hospitals (Rizzoli Orthopedic Institute - 0012688; 13/03/2013 coordinator of the study; University Hospital of Bologna − 189/2013/O/Oss; 29/10/2013; Hospital of Reggio Emilia). All procedures performed in studies involving human participants were in accordance with the 1964 Helsinki declaration and its later amendments or comparable ethical standards. Written informed consent was given by all participating patients or their legal representatives.

## Results

**Figure 1:**
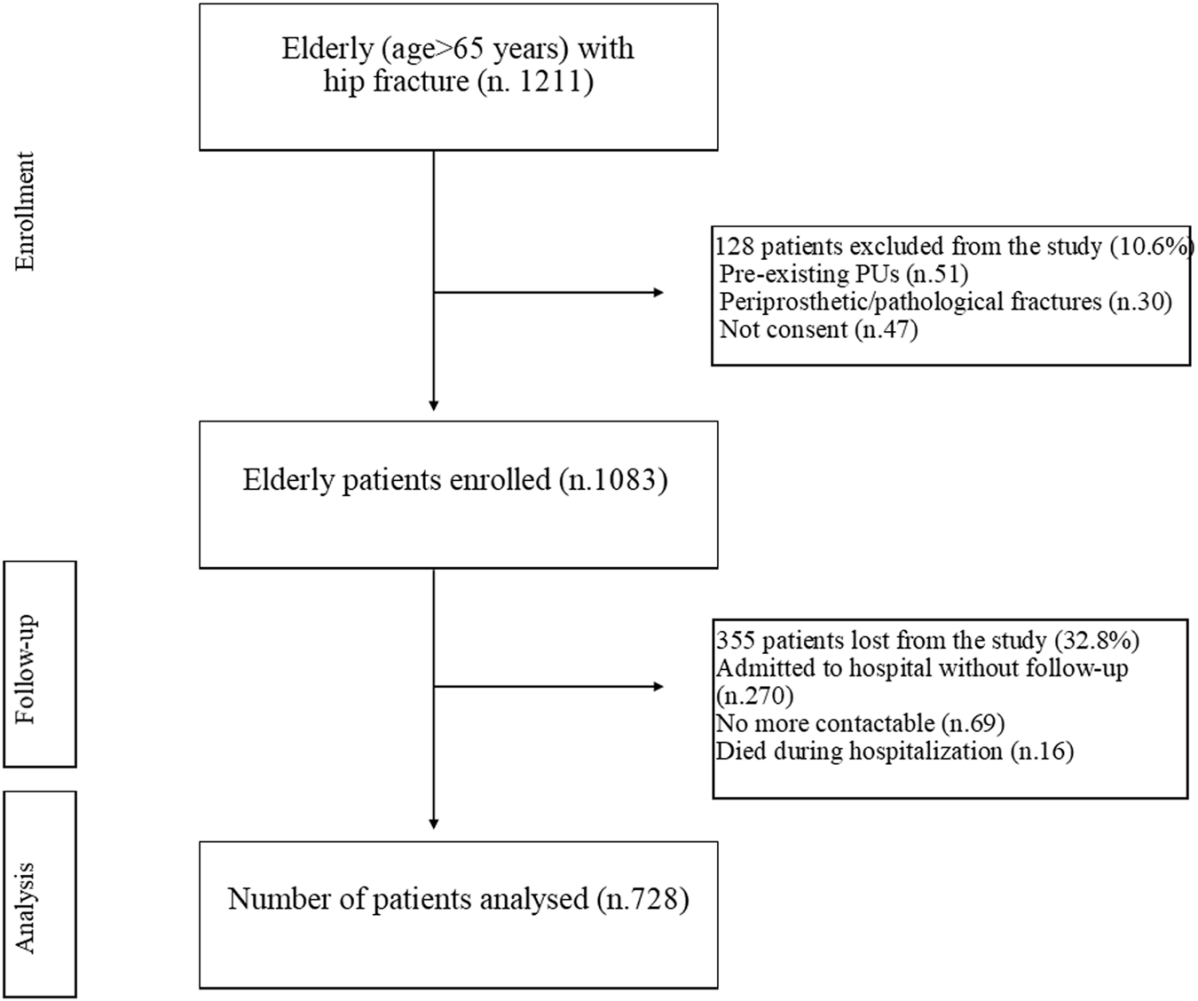
Enrollment process.

Of the 1211 eligible patients, 128 did not fulfil the inclusion criteria. In the excluded patients. 51 (39.8%) presented pre-existing pressure ulcers at hospital admission (exclusion criteria for the primary outcome), 30 (23.4%) had a diagnosis of periprosthetic or pathological fractures, and 47 (36.7%) did not agree to participate in the study (Fig. 1). The final population was 1083 patients. The planned one-year follow-up was reached in 728 patients. Among the excluded patients, 270 (76%) were admitted to one of the involved hospitals, which did not collect data at 12 months after surgery due to organizational problems, 69 (19.4%) were no more contactable and 16 (4.5%) died during hospitalization (Fig. 1). Comparing characteristics at hospital discharge of those patients included in the final cohort and those lost to one-year follow-up, no statistically significant differences emerged.

Baseline and age adjusted characteristics of study participants are shown in Table 1.

**Table 1:** Characteristics of hip fracture patients according to survival status within one-year.

Variables	Patients alive (n.607)	Patients died (n.121)	All patients (n.728)	P -value	Age adjusted P-value
***Preoperative variables***					
Age (mean, SD)	82.8 (7.8)	88.4 (6.8)	83.8 (7.9)	<0.001	—*
Female gender - n. (%)	475 (78.3)	90 (74.4)	565 (77.6)	0.403	0.176
Patients coming from nursing homes (vs home) - n. (%)	48 (7.9)	11 (9.1)	59 (8.1)	0.715	0.703
Patients with other fractures at presentation - n (%)	44 (7.2)	11 (9.1)	55 (7.6)	0.571	0.768
Pre-fractures ADL score^a^	0 (2)	2.5(4)	0 (3)	<0.001	<0.001
Charlson Index Score^b^	2 (2)	2 (3)	2 (2)	<0.001	<0.001
Average baseline Hb level (g/dl)	12.6 (2.1)	12.3 (2.2)	12.6 (2.2)	0.005	0.124
Patients with normal physical condition - n (%)	427 (71.4)	79 (65.8)	506 (70.5)	0.229	0.561
Patients with pressure ulcers at presentation - n (%)	23 (3.8)	11 (9.1)	34 (4.7)	0.018	0.041
Patients with femoral neck fracture (vs. trochanteric) - n (%)	291 (47.9)	56 (46.3)	347 (47.7)	0.766	0.424
***Surgery variables***					
Patients with Osteosynthesis surgery (vs. arthro- or endoprothesis) - n (%)	331 (54.5)	67 (55.4)	398 (54.7)	0.920	0.442
Lenght of surgery (minutes)	60 (33)	61 (30)	60 (33)	0.362	0.508
Number of hours from arrival in the ED to surgery	29 (24)	29 (30)	29 (25)	0.596	0.533
Patients operated on within 48 hours - n (%)	469 (77.3)	89 (73.6)	558 (76.6)	0.410	0.421
***Postoperative variables***					
Percentage of decrease in Hb with respect to the initial values	24.6 (15.1)	21.3(18.1)	23.7 (15.9)	0.001	0.023
Patients with lack recovery of ambulation - n (%)	217 (35.7)	71 (58.7)	288 (39.6)	<0.001	0.001
Patients with disorientation - n (%)	328 (54)	96 (79.3)	424 (58.2)	<0.001	0.002
Patients with pressure ulcers - n (%)	159 (26.4)	47 (39.5)	206 (28.5)	0.004	0.019
Percentage of days with pain ≥4	12.5 (25)	11.1 (24)	12.5 (25)	0.580	0.942
Patients with urinary infection - n (%)	84 (13.8)	20 (16.5)	104 (14.3)	0.477	0.971
Patient admitted to an orthogeriatic ward - n (%)	178 (29.3)	37 (30.6)	215 (29.5)	0.827	0.707
Percentage of days with the presence of an informal caregiver for at least half day	90.9 (38.5)	83.3 (58.3)	90 (41.7)	0.02	0.012
Length of stay (days)	9 (3)	9 (4)	9 (4)	0.131	0.083
Patients compliant with post-discharge pathway - n (%)	47 (7.8)	10 (17.2)	57 (8.6)	0.019	0.018

The data are reported as median (IQR) or percentages.

Patients with pressure ulcers evaluated for 722 patients; Post-discharge pathway evaluated for 664 patients.

SD, Standard Deviation; Hb, Hemoglobin; ED, Emergency Department.

*Value is not age adjusted.

^a^Activity Daily Living = from 0, independent on activities of daily living, to 7, dependent.

^b^Charlson Index = from 0, no significant comorbidity, to 33, severe comorbidity.

During one year follow up 121 patients (16.6%) died. Comparing the occurrence of death between males (14.3%) and females (11.9%), no statistically significant differences were found.

### Predictive factors of one-year mortality

Eleven factors were significantly related to the outcome according to the univariate analysis (Table 1). These did not include any of the variables directly connected to surgery. Among infections, only urinary infection was included in the data analysis, having an overall occurrence of 14.3%. Surgical wound and pulmonary infections were respectively 0.1% (1 case) and 1.1% (8 cases), thus they were not included in the statistical analysis. The multivariate analysis allowed us to identify a total of five independent predictive factors (Table 2). Being older (OR = 1.094, 95% CI = 1.057–1.132), having a higher baseline Charlson Index (OR = 1.257, 95% CI = 1.114–1.418) and ADL (OR = 1.259, 95% CI = 1.143–1.388) score, presenting hospital-acquired PUs (OR = 1.579, 95% CI = 1.002–2.489) and experiencing lack recovery of ambulation (OR = 1.736, 95% CI = 1.115–2.703), were found to be statistically significant predictive factors of one year mortality after surgery.

**Table 2:** Factors affecting one-year mortality: multivariate analysis.

Predictive factor	OR	95%CI	P-value
Age (for each additional year)	1.094	1.057; 1.132	0.0005
Charlson Index Score (for each unit increase)	1.257	1.114; 1.418	0.0005
Pre-fractures ADLs score (for each unit increase)	1.259	1.143; 1.388	0.0005
Hospital acquired pressure ulcers (for presence)	1.579	1.002; 2.489	0.049
Lack recovery of ambulation (for presence)	1.736	1.115; 2.703	0.015

OR, Odds RATIO; CI, Confidence Interval; ADLs, Activities of Daily Living.

**Figure 2:**
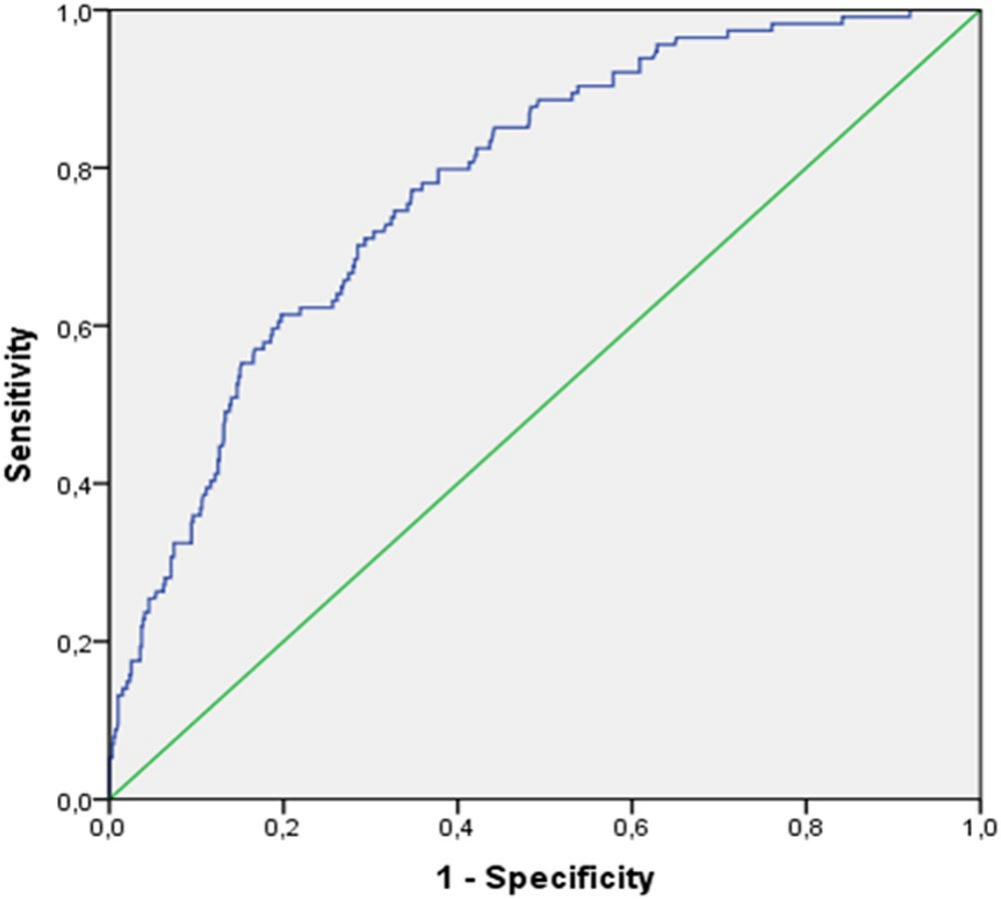
ROC Curve of the predictive model of one-year mortality in patients with hip fractures. AUC 0.780 (CI95% 0.0737–0.824).

The ROC curve (Fig. 2) constructed using the probability of verifying the outcome derived from the model which resulted from the multivariate analysis had an area under the curve (AUC) of 0.780 (95% CI: 0.737–0.824).

## Discussion

In the present study of 728 patients who underwent surgery for hip fractures, 121 (16.6%) died within one-year from surgery. The overall death occurrence observed in our study was broadly lower when compared with most previous studies^4–10,13,28^. Nevertheless, the heterogeneity of the population studied, the differences in care standards and the lack of consistency of the study designs and statistical analysis used to determine excess mortality, could make the comparison difficult.

In our sample, three statistically significant preoperative variables which predicted a greater risk of mortality at 12 months following hip fracture surgery, were identified. According to previous studies^4,7,8,11,12,14–16^, these included advanced age, pre-fracture dependence in ADLs and a higher number of baseline comorbidities. For each one-year increase in age, there was a 9.4% increase in risk of dying within 12 months from surgery. This is probably due to the elderly increased vulnerability to stressors resulting in decreased physiological reserves and deregulation of multiple systems.

In the same way, the one-year mortality risk was increased by 26% for each point increase on both the Charlson Index and ADLs score. These may be associated with patient’s inadequate rehabilitation resources. Indeed, elderly frailty has been previously related to increased susceptibility to various adverse health outcomes, such as death^29,30^.

Unlike previous studies reporting an association between one-year mortality and variables related to surgical intervention, such as time to surgery^6,8,11–15,18^, this relation was not found in the present study. This is probably since almost 80% of our patients underwent early surgery (within 48 hours from the trauma), as recommended by International guidelines^31,32^.

With regard to postoperative variables, the development of hospital-acquired PUs was found to be a predictive factor of one-year mortality. Again, this could be explained with a poorer physical condition of the patients who developed PUs during hospitalization. This hypothesis is supported by previous results^10^, who showed that the presence of PUs was independently related to reduced functional recovery. Mariconda and colleagues^8^ found that surgical complications, including disorientation, pressure ulcer and anaemia, represented the basic predictors of one-year mortality.

Another post-operative variable that emerged as a significant predictor of one-year mortality was failure to recover ambulation. Upon discharge from hospital, 40% of the studied patients were not able to recover independent ambulation. This is in line with results of Voctheloo and colleagues^33^, who found that more than 50% of patients did not return to previous mobility levels at 12 months following hip fracture. Consistently with our results, Siu and colleagues^22^ showed reduced survival rates following delayed ambulation, probably because of the potentially adverse effect of immobility.

Among the other postoperative variables tested in the present study, anaemia and disorientation were significant when univariate analysis was performed, but this significance disappeared when adjusting for baseline covariates at the multivariate analysis. This is supported by a recent systematic review and meta-analysis by Hamilton and colleagues^34^, which concluded that in studies on geriatric populations where specific confounders, such as age, gender, comorbidities, initial cognitive status and type of surgery, were tested, disorientation was no longer a predictive factor of mortality. Pulmonary and wound infections proved to be a rare complication in clinical practice, thus it could not be analysed from a statistical point of view, as supported by Mariconda and colleagues^8^. Only urinary tract infections were analysed but were not found to be significant.

The model analysed using the ROC curve showed good predictability for mortality (AUC included between 0.7 and 0.8), in line with previous instruments developed for predict one-year mortality in geriatric patients, such as  the modified Canadian Study of Health and Aging Frailty Index^35^.

### Strengths and limitations

Two of the strengths of this study are its prospective design and sample size. A cohort of 728 patients with hip fractures who were consecutively admitted to two large acute hospitals during the one-year study period, were analysed. Furthermore, to our knowledge, a study analysing such a wide range of peri-operative variables has not been previously reported.

Nevertheless, some limitations affect the study. First of all, almost 33% of the originally included patients were lost to one-year follow-up. Nevertheless, no statistically significant difference was noted between the included and lost patients with regard to clinical data at hospital discharge.

Another important limitation of this study was related to the follow-up methodology; the assessment of mortality was based on calling patient’s family by phone, thus resulting in some missed information, such as the cause of death. Moreover, in many cases the exact date of death was not assessed, thus preventing the possibility to perform some statistical analysis, such as Cox model regression and Kaplan Meier survival curve.

Additionally, some variables, such as disorientation, were not assessed using validated measurements but with a clinical evaluation; nevertheless, this was more consistent with the daily clinical practice. Moreover, although several covariates were included in the model to adjust for differences in patient characteristics, unmeasurable or unmeasured variables that might affect the likelihood of one-year mortality may not have been considered. Finally, no information was available on pre-fracture cognitive status.

## Conclusions

In this prospective cohort study of 728 patients who underwent surgical treatment for hip fractures, we found the predictive factors for one-year mortality included older age, a higher number of comorbidities, a higher pre-fracture dependence in ADLs, the presence of hospital acquired pressure ulcers and lack of recovery ambulation. These results seem to suggest frailty as the main predictor of one-year mortality. Frail patients need careful long-term follow-up with attention to comorbidity and disability; at the same time, healthcare organizations might implement and/or improve early ambulation.
